# Zielgerichtete Therapie beim metastasierten Mammakarzinom – welche molekularen Tests sind notwendig?

**DOI:** 10.1007/s41974-020-00141-z

**Published:** 2020-06-02

**Authors:** Marcus Schmidt

**Affiliations:** Abteilung für Molekulare Onkologie, Klinik und Poliklinik für Geburtshilfe und Frauengesundheit, Universitätsmedizin der Johannes Gutenberg-Universität Mainz, Langenbeckstr. 1, 55131 Mainz, Deutschland

**Keywords:** Mammakarzinom, Programmed cell death ligand 1 (PD-L1), Breast cancer (BRCA)1/2, Phosphatidylinositol-4,5-bisphosphate 3-kinase catalytic subunit alpha (PIK3CA), Präzisionsonkologie, Breast neoplasms, Programmed cell death ligand 1 (PD-L1), Breast cancer (BRCA)1/2, Phosphatidylinositol-4,5-bisphosphate 3-kinase catalytic subunit alpha (PIK3CA), Precision oncology

## Abstract

In den letzten Jahren ist die zielgerichtete Therapie beim Mammakarzinom immer mehr in den Fokus gerückt. Neben den Hormonrezeptoren und dem humanen epidermalen Wachstumsfaktor 2 (HER2) sind derzeit für die Festlegung einer gezielten Therapie vor allem der immunhistochemische Nachweis des „programmed cell death ligand 1“ (PD-L1) bei fortgeschrittenen triple-negativen Mammakarzinomen sowie der Nachweis von Mutationen im Breast-cancer-1(BRCA1)- oder BRCA2-Gen in der Keimbahn der Patientinnen und von Mutationen im Phosphatidylinositol-3-Kinase(PI3K)-Weg relevant.

## Abstract (en)

In recent years, targeted therapy for breast cancer has received increasingly more attention. In addition to hormone receptors and human epidermal growth factor 2 (HER2), the immunohistochemical detection of programmed cell death ligand 1 (PD-L1) in advanced triple-negative breast cancer and the detection of mutations in the breast cancer 1 (BRCA1) or BRCA2 gene in the patient’s germline and mutations in the phosphatidylinositol 3 kinase (PI3K) pathway are currently relevant when making decisions regarding targeted therapy.

Zielgerichtete Therapien werden beim Mammakarzinom seit vielen Jahren verwendet. Neben dem Nachweis der Hormonrezeptoren spielt der humane epidermale Wachstumsfaktor 2 (HER2) bei der Therapie des frühen wie auch des fortgeschrittenen Mammakarzinoms eine entscheidende Rolle [1, 2]. Darüber hinaus hat eine verbesserte molekularbiologische Charakterisierung des Mammakarzinoms in der letzten Dekade zu neuen Angriffspunkten für eine gezielte Therapie geführt [3]. Dies erweitert die Möglichkeiten der zielgerichteten Therapie beim Mammakarzinom.

Beim sog. triple-negativen Mammakarzinom (TNBC), das negativ für den Östrogenrezeptor (ER), den Progesteronrezeptor (PR) und HER2 ist, ist in den letzten Jahren die Bedeutung des Immunsystems immer mehr in den Fokus des Interesses gerückt. Beim TNBC werden deutlich mehr somatische Mutationen und Neoantigene nachgewiesen als bei anderen molekularen Subtypen, was für eine erhöhte Immunogenität spricht [4]. Tatsächlich konnte gezeigt werden, dass sowohl Transkripte von Immunzellen als auch tumorinfiltrierende Lymphozyten (TIL) ihren stärksten prognostischen und prädiktiven Einfluss bei TNBC haben [5–7]. Mit dem Immuncheckpointinhibitor (ICPi) Atezolizumab konnte ein verbessertes progressionsfreies Überleben (PFS; 7,5 vs. 5 Monate; Hazard Ratio [HR] 0,62) sowie ein verlängertes Gesamtüberleben (OS; 25 vs. 15,5 Monate; HR 0,629) bei fortgeschrittenen TNBC mit Positivität für den *„programmed cell death ligand 1“ (PD-L1)* gezeigt werden [8].

Ein weiterer prädiktiver Marker beim fortgeschrittenen Mammakarzinom ist der Nachweis von Mutationen im Breast-cancer-1(*BRCA1*)*-* oder *BRCA2*-Gen in der Keimbahn der Patientinnen. Bei Nachweis einer BRCA-Mutation können Inhibitoren der Poly(ADP-Ribose)-Polymerase (PARP) eingesetzt werden. Bei BRCA-mutierten Tumoren, die einen Defekt in der homologen Rekombination, die zur fehlerfreien Reparatur von DNA-Doppelstrangbrüchen dient, aufweisen, führt dies zur sogenannten synthetischen Letalität [9]. Durch die Blockade dieser beiden Reparaturmechanismen können die Tumorzellen DNA-Schäden nicht mehr effizient reparieren. Die PARP-Inhibitoren Olaparib (8,0 vs. 4,2 Monate; HR 0,58) und Talazoparib (8,6 vs. 5,6 Monate; HR 0,54) verlängern das PFS bei Patientinnen mit einer BRCA-Mutation [10, 11]. Für Olaparib ist zwischenzeitlich auch eine OS-Verlängerung bei Patientinnen, die in der ersten Therapielinie behandelt wurden, nachgewiesen [12].

Bei Hormonrezeptor-positiven fortgeschrittenen Mammakarzinomen spielt der Phosphatidylinositol-3-Kinase(PI3K)-Weg eine wesentliche Rolle. Patientinnen mit Mutationen im *PIK3CA*-Gen zeigen ein signifikant verlängertes PFS (11 vs. 5,7 Monate; HR 0,65), wenn sie zusätzlich zu Fulvestrant noch mit dem PI3K-Inhibitor Alpelisib behandelt werden [13]. „Downstream“ von PI3K liegt die Kinase *AKT*. Das PFS (10,3 vs. 4,8 Monate; HR 0,58) konnte durch Hinzunahme des AKT-Inhibitors Capivasertib bei Hormonrezeptor-positiven Patientinnen in der FAKTION-Studie signifikant verlängert werden [14]. Auch bei fortgeschrittenem TNBC führen AKT-Inhibitoren in der PAKT-Studie zu einem verbesserten PFS (5,9 vs. 4,2 Monate; HR 0,74) und zeigen sogar eine Verlängerung des OS (19,1 vs. 12,6 Monate; HR 0,61; [15]). Diese Veränderungen waren bei Tumoren mit Alterationen im *PIK3CA/AKT1/PTEN*-Weg am deutlichsten (PFS 9,3 vs. 3,7 Monate; HR 0,14), sodass eine genauere molekularbiologische Charakterisierung in Zukunft immer wichtiger wird.

Beim fortgeschrittenen Mammakarzinom können Mutationen im Östrogenrezeptor *(ESR1)* nachgewiesen werden, die unter einer Behandlung mit Aromatasehemmern selektiert werden. In der SOFEA-Studie wiesen 39,1 % und in der PALOMA-3-Studie 25,3 % eine *ESR1-Mutation* auf. Mammakarzinome mit einer solchen *ESR1-Mutation* sprechen in retrospektiven Analysen prospektiver Studien besser auf eine Therapie mit Fulvestrant als auf einen Aromatasehemmer an [16]. Die Hazard Ratio lag zwischen 0,43 (PALOMA-3) und 0,52 (SOFEA).* HER2-Mutationen* treten unabhängig von einer HER2-Überexpression oder -Amplifikation in 2,4 % auf, wobei die Rate bei lobulären (7,2 %) höher ist als bei duktalen (1,6 %) Mammakarzinomen [17]. In einer kleinen Phase-II-Studie hatten stark vorbehandelte Patientinnen mit einer HER2-Mutation ein progressionsfreies Überleben (PFS) von 16 Wochen unter dem Tyrosinkinaseinhibitor Neratinib. Ähnlich wie bei einer ESR1- oder einer PK3CA-Mutation kann auch eine *HER2-Mutation* aus zirkulierender Tumor-DNA (ctDNA) nachgewiesen werden, was die Praktikabilität verglichen mit der Mutationsanalyse aus einer Biopsie einer Metastase verbessert.

Seit Langem besteht eine große Hoffnung in einer verbesserten molekulargenetischen Charakterisierung, die durch den Nachweis von therapierbaren („actionable“) Mutationen zu einer Präzisionsmedizin („precision medicine“) mit verbesserter Prognose bei fortgeschrittenen Krebserkrankungen führen soll. Das Konzept der Präzisionsmedizin ist überzeugend, allerdings liegt bislang erst eine prospektive, randomisierte Studie in der Onkologie vor. In der SHIVA-Studie untersuchten Le Tourneau und Mitarbeiter 741 fortgeschrittene Malignome (~20 % Mammakarzinome) und konnten bei 40 % molekulare Alterationen finden [18]. Die Patienten wurden dann randomisiert zwischen einer Standardtherapie und einer gezielten Therapie der molekularen Alterationen. Das PFS konnte allerdings durch die gezielte mutationsspezifische Therapie nicht signifikant verbessert werden (PFS 2,3 vs. 2,0 Monate; HR 0,88; *P* = 0,41), sodass die Autoren aus ihren Ergebnissen schlussfolgerten, dass ein Einsatz von gezielten Therapien außerhalb der Zulassung nicht unterstützt werden kann. Dessen ungeachtet sind weitere mögliche Angriffspunkte für gezielte Therapien beim fortgeschrittenen Mammakarzinom, die allerdings in weniger als 1 % der Mammakarzinome zu finden sind, zum einen der Nachweis einer *Mikrosatelliteninstabilität (MSI)*, die zu einer deutlich erhöhten Rate an Mutationen führt [19], und zum anderen eine *Neurotrophe-Tyrosinrezeptorkinase*(*NTRK)-Genfusion*, die zumeist bei sekretorischen Mammakarzinomen beobachtet wird [20]. Sowohl für Malignome mit *NTRK-*Genfusion als auch mit *Mikrosatelliteninstabilität* können Therapien tumoragnostisch mit Erfolg eingesetzt werden [21, 22], wobei allerdings auch hier bislang noch randomisierte Evidenz aussteht.

Insgesamt haben die verbesserte molekularbiologische Charakterisierung und die Entwicklung neuer molekularer Tests, von denen der Nachweis von *PD-L1* sowie Mutationsanalysen von *BRCA* und *PIK3CA* bereits als Standard betrachtet werden können, die therapeutischen Optionen beim fortgeschrittenen Mammakarzinom deutlich erweitert.
